# Antiinfective properties of ursolic acid-loaded chitosan nanoparticles against Staphylococcus aureus

**DOI:** 10.3906/kim-2104-13

**Published:** 2021-10-19

**Authors:** Fatemeh GHASEMZADEH, Ghasem D. NAJAFPOUR, Maedeh MOHAMMADI

**Affiliations:** 1 Biotechnology Research Laboratory, Faculty of Chemical Engineering, Babol Noshirvani University of Technology, Babol Iran

**Keywords:** Ursolic acid-loaded chitosan nanoparticle, *Staphylococcus aureus *bacteria, biofilm

## Abstract

The present study aimed to synthesize ursolic acid-loaded chitosan nanoparticles (UA-Ch-NPs) as an antiinfective agent against 21 *Staphylococcus aureus* isolates. The UA-Ch-NPs were synthesized by a simple method and then characterized by TEM, FTIR, DLS-zeta potential, and XRD analyses. According to the characterization results, highly dispersed spherical nanoparticles with a mean diameter of 258 nm and a zeta potential of + 40.1 mV were developed. The antibacterial properties of UA-Ch-NPs were investigated and their inhibitory effect on biofilm formation was demonstrated by AFM. Finally, the expression levels of icaA and icaD were measured using real-time PCR. Results indicated that the minimum inhibitory concentration (MIC) of UA and UA-Ch-NPs against *S. aureus *was 64 and 32 µg/mL, respectively. The treatment of bacterial cells with UA-Ch-NPs significantly decreased the expression of icaA and icaD genes which are engaged in biofilm formation. Our results indicated that UA-Ch-NPs could be a promising material for antibacterial and antibiofilm applications.

## 1. Introduction

*Staphylococcus aureus* as a Gram-positive bacterium causes several infections including nosocomial infections. *S. aureus* infections have been shown to be highly variable. For instance, *S. aureus* causes acute diseases, like bacteremia and severe chronic infections [1]. In recent years, *S. aureus* has represented a serious challenge because of its ability for developing resistance against currently available antibiotics. This resistance is generally linked to the capability of the bacteria to form biofilm. *S. aureus* strains that are isolated from mastitis in ruminants, for example, produce slime in vivo, resulting in a remarkably higher capacity of colonization in comparison with nonslime producing variants. As a result of biofilm formation in mastitis isolates, susceptibility to antibiotics is reduced, which is the consequence of the reduced antibiotic diffusion through the biofilm matrix and a reduction of the metabolic activity of bacteria inside the biofilm [2]. Thus, a new generation of antibacterial agents is urgently needed to fight *S. aureus*.

Ursolic acid (UA, 3β-hydroxyurs-12-en-28-oic acid,) as a natural pentacyclic triterpenoid carboxylic acid is found in various plants. This agent is highly potential to provide defense against certain pathogens such as *E. coli* and *S. aureus* [3,4]. UA has been indicated to have various effects on some microorganisms, characterizing it as an antibiotic drug. Kozai et al. [5] demonstrated that UA managed to inhibit insoluble glucan synthesis in* Streptococcus mutans*. Moreover, UA has been shown to fight against *S. sobrinus* and *S. mutans,* with a minimum inhibitory concentration (MIC) of 2.0 μg/mL [6]. Furthermore, the antibacterial properties of UA have been discovered on other human bacterial pathogens, including* S. pneumonia*, methicillin-sensitive, and methicillin-resistant *Bacillus cereus*, *Staphylococcus aureus*, and* Pseudomonas aeruginosa* [7]. 

Despite its ability to kill bacteria, UA has low water solubility which limits its clinical applications. Therefore, different methods were proposed for improving UA antibacterial activity. Some nano-drug delivery systems have been used for increasing drug solubility [8, 9]. In this regard, some natural water-soluble polymers, like alginate, chitosan, or poly(lactic-co-glycolic acid) have been widely employed in drug delivery systems owing to their biodegradability and biocompatibility features [10–13]. 

Chitosan (Ch) is a natural polysaccharide polymer; it is a deacetylated chitin derivative. Thanks to its low toxicity and high biocompatibility, this biological material has been widely used in many biomedical applications [14,15]. Ch has potency for drug delivery through the paracellular pathway. Jin et al. [10] loaded UA in Ch nanoparticles to study their antiangiogenesis activity in tumors through in vitro and in vivo investigations. Although synthesis of UA-loaded Ch nanoparticles and their antitumor properties have been reported, to the best of authors’ knowledge, there is no study concentrating on the antiinfective properties of the UA-loaded Ch nanoparticles. Therefore, this study aims to investigate the antiinfective properties of ursolic acid-loaded chitosan nanoparticles (UA-Ch-NPs) against *S. aureus*. It was assumed that UA-Ch-NPs could overcome the solubility of UA and improve its efficacy. The biological applications, such as growth-inhibitory effects and biofilm formation prevention activity of this agent were evaluated as well.

## 2. Materials and methods

### 2.1. Materials

Chitosan (M_n_ = 1600 determined by the supplier) and ursolic acid (98.6%) were purchased from Sigma-Aldrich Co., Germany. Ethyl-(3-3-dimethylaminopropyl) and N-hydroxy-succinimide (NHS) were purchased from Beyotime Institute of Biotechnology, China. *S. aureus *ATCC 25923 was supplied by Pasteur Institute, Iran. Penicillin and Ciprofloxacin were obtained from Jaber Ebne Hayyan Pharmaceutical Co., Iran. Ceftiofur, erythromycin, tetracycline, trimethoprim, ampicillin, chloramphenicol, and gentamicin were obtained from Pharmacia & Upjohn, Belgium.

### 2.2. Synthesis of UA-Ch-NPs

UA-Ch-NPs were synthesized as described previously [10]. In brief, 32 mg of Ch was dissolved in 5 mL 1% glacial acetic acid. Then, 10 mg UA, 30 mg ethyl-3-(3-dimethylaminopropyl), and 8 mg N-hydroxysuccinimide (NHS) were mixed with the obtained solution. It was then vortexed overnight at ambient temperature. Then, the sample was centrifuged for 20 min at 11,000 rpm. The harvested pellets were UA-Ch-NPs that were lyophilized for prolonged storage.

### 2.3. Characterization of UA-Ch-NPs

To confirm the successful production of UA-Ch-NPs, Fourier transform infrared (FTIR) analysis was conducted using a FTIR spectrophotometer (Perkin Elmer Spectrum RXI). This experiment was carried out for UA, Ch, and UA-Ch-NPs samples. Transmission electron microscopy (TEM; Zeiss, Oberkochen, Germany) was used to study the morphology of the prepared samples and Digimizer image analysis software (version 5.3.4) was used to measure the mean size of the nanoparticles. The X-ray diffraction (XRD) analysis was also performed using an INEL Equinox 3000 diffractometer with the CuKα (λ = 1.5406 Å) radiation. The size and surface charge of the produced UA-Ch-NPs were analyzed using DLS-zeta potential analyzer (NanoPlus-Micromeritics, USA). The surface zeta potential of UA-Ch-NPs was calculated using a dynamic light scattering (DLS) instrument (Brookhaven 90 Plus/BI-MAS). 

### 2.4. Antibiotic susceptibility testing 

*S. aureus *ATCC 25923 was purchased from Pasteur Institute of Iran. A total of 21 *S. aureus *isolates obtained from bovine mastitis were used in this study; the isolation was carried out in the pharmaceutical laboratory, Shahid Beheshti University of Medical Sciences, Tehran, Iran. The disc diffusion approach was used to carry out antibiotic sensitivity testing (AST) against ceftiofur, erythromycin, tetracycline, trimethoprim, penicillin, ampicillin, chloramphenicol, gentamicin, and ciprofloxacin. Firstly, inoculation of the Mueller–Hinton agar Petri dishes with half-McFarland of bacteria was done, followed by the placement of antibiotic discs right onto the agar. The diameters around the discs were determined after 24 h of incubation. The sensitivity to UA-Ch-NPs was assessed based on the inhibition zone diameter, based on the Clinical and Laboratory Standards Institute (CLSI) protocol [16].

### 2.5. Minimum inhibitory concentration (MIC) 

The MIC value of all pharmaceutical agents was measured by microdilution assay in 96-well plates. Accordingly, 100 µL of sterilized Mueller–Hinton broth (MHB) was added to each well, followed by treating each well separately with serial dilutions of UA and UA-Ch-NPs. The microbial cultures were then adjusted to the turbidity of a 0.5 McFarland standard. The 0.2 mg/mL p-iodonitrotetrazolium chloride (INT) was mixed with each sample. Finally, following incubation at 37 °C for 24 h, the lowest concentration before the color change was determined as the MIC value. 

### 2.6. Bacterial adhesion to hydrocarbons test

The bacterial adhesion to hydrocarbons test was used for measuring the relative hydrophobicity of various bacterial strains following the work by Subbiahdoss et al. [17]. In short, bacterial suspension was mixed with n-decane (hydrocarbon phase) at a 1:1.2 ratio, followed by vortexing for 2 h. After the separation of the obtained emulsion, the optical density of the aqueous phase was measured at 600 nm (OD_600_). The bacteria adhesion to the oil phase was quantified through calculation of the ratio between the optical density prior to and after mixing. Finally, the hydrophobicity degree was categorized into three classes: > 70%, 30%–70%, and < 30% for strong, moderate, and low hydrophobicity, respectively. 

### 2.7. Biofilm formation assay 

To evaluate the biofilm formation ability of the samples, bacterial suspension was initially added to sterile tryptic soy broth (TSB) supplemented with 1% glucose and then seeded into 96-well flat-bottom sterile polystyrene microplate. The broth without bacteria and the inoculated broth without the nanoparticles were considered as negative and positive controls, respectively. The samples were incubated at 37 °C for 24 h. Biofilm stains and the culture medium were removed and washed with phosphate buffered saline (PBS). Afterward, 100 μL gentian violet, 1%, was added and incubated at room temperature for 5 min. The samples were then washed with distilled water to wipe up the excess gentian violet. Eventually, 200 μL glacial acetic acid (33% (w/v)) was added to each well. After 20 min, the ELISA reader (BioTek, Citation3) was used to read the absorbance of each sample at 640 nm [18]. 

The OD_640_ values were employed for comparative analysis and semi-quantitative classification of the biofilms produced by the bacterial strains based on the approach presented by Stepanovic et al. [19]. Briefly, the cut-off OD (ODc) was set as three standard deviations above the mean OD of the negative control, and classification of strains was as follows: OD > 4 × ODc = strong biofilm producer; 2 × ODc < OD < 4 × ODc = moderate biofilm producer; ODc < OD < 2 × ODc = weak biofilm producer; and OD < ODc = poor biofilm producer.

Atomic force microscopy (AFM, Thero-microscopes, CP Research, USA) was used to study the biofilm formation in a contact mode of 10 × 10 µm^2^. The silicon nitride tips used in the measurements were irradiated with UV for 15 min in the air to remove any organic contaminants. The tip curvature radius was less than 10 nm. The force constant and oscillation frequency were 0.03 N/m and 255 kHz, respectively.

### 2.8. Determination of bacterial growth curves

OD measurements for the development of bacterial growth curves were performed by determining the absorbance at a wavelength of 620 nm. Bacteria were cultured in Costar 96-well flat-bottom plate and treated with different concentrations of UA and UA-Ch-NPs (i.e., MIC, 0.5 MIC, and 2 MIC). The OD_620_ values of bacterial cultures were measured at 15 min intervals for 24 h and each one was read 3 times. 

### 2.9. Real-time quantitative reverse transcription-polymerase chain reaction (RT-qPCR)

Total RNA was extracted from treated and untreated *S. aureus *using commercial RNA extraction kits (Qiagen, Germany) according to manufacturer instructions. The equal quantity of RNA extracted from each group of bacteria was treated by 5 μL of DNase 1 (Qiagen, Germany) for the probable presence of contaminating DNA. The RNA samples were then checked for the 16S rRNA gene as an internal standard using below 16S primers. According to the manufacturing protocol (TaKaRa, Japan), total RNA (2 μg) was used for cDNA synthesis. The reaction was carried out under the following conditions: one cycle at 37 °C for 15 min for reverse transcription, 94 °C for 30 s for the inactivation of enzymes up to 10 μL, and without hot commencement [20]. The RT-qPCR reactions were performed using SYBR Green PCR Master Mix (Qiagen, Germany) and ABI 7000 instrument (Applied Biosystems, Italy). The used primers were as follows: icaA Forward; 5-ACA CTT GCT GGC GCA GTC AA-3, icaA Reverse; 5-TCT GGA ACC AAC ATC CAA CA-3, icaD Forward; 5-ATG GTC AAG CCC AGA CAG AG-3, icaD Reverse; 5-TGA ACT TAT TCC ACC GCC TTT A-3, Bap Forward; 5- ATA CTG ATG GCG ATG GTA-3, Bap Reverse; 5-ACT GTG TCT TCT GTT GTA AT-3, 16S forward; 5-ACT GGG ATA ACT TCG GGA AA-3 and 16s reverse; 5-CGT TGC CTT GGT AAG CC-3. The PCR mixture’s final volume was 25 μL with 12.5 μL of the master mix, 1 μL each of forward primer and reverse primer, 1 μL of cDNA, and 9.5 μL of free water. The cycling conditions were: 1 cycle at 95 °C for 10 min, and then 40 cycles at 95 °C for 20 s, and 60 °C for 40 s. Finally, to draw the melting curve, the samples were incubated at 95 °C for 15 s, 60 °C for 30 s, and 95 °C for 15 s.

### 2.10. Data analysis

One-way ANOVA was used for the statistical analysis of the experimental data, and results were presented as means ± SD. Also, p < 0.05 was considered statistically significant to verify the variations in the results due to the effect of different treatments. 

## 3. Results 

### 3.1. Characterization of UA-Ch-NPs

**Figure 1:**
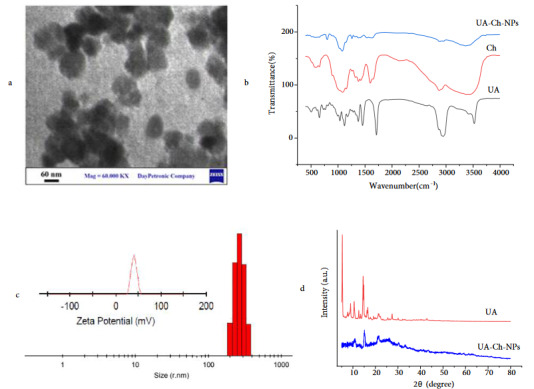


The TEM image of the synthesized UA-Ch-NPs is shown in Figure 1a. As shown, spherical nanoparticles with an almost uniform particle size of 75 nm were obtained. The FTIR spectra of UA, Ch, and UA-Ch-NPs are presented in Figure 1b. A unique characteristic peak in the FTIR spectrum of UA at 3520 cm^–1^ is observed, which is identified as the O–H stretching vibration. The peak at 2940 cm^–1^ corresponds to the C-H stretching vibrations in methylene groups. The bands at 1720, 1380, 1463, and 1090 cm^–1 ^are related to the C=O stretching vibrations in the carboxylic acid group, C-H groups, C=C stretching vibrations, and C-O stretching vibrations in secondary alcohols, respectively [21]. Ch also showed some characteristic peaks in the FTIR analysis. The presence of O-H, N-H, and intramolecular hydrogen bonds in Ch was confirmed by stretching vibrations around 3200–3600 cm^–1^. The peaks at 2920 and 2870 cm^–1^ are related to symmetric and asymmetric stretching vibrations of C-H groups, respectively. The bands at 1650, 1590, and 1350 cm^–1^ are associated with the C=O stretching vibrations in type I amide, the N-H bending vibrations in type II amide, and the C-N stretching vibrations in type III amide, respectively. The peaks at 1450 and 1360 cm^–1^ are attributed to the C-H bending and symmetrical vibrations in CH_2_ and CH_3_. The absorption bands at 1150 and 1000 cm^–1^ are related to asymmetric vibrations of C-O-C bridges and C-O stretches, respectively [22,23]. The changes in the intensity of these peaks and the formation of new peaks in the FTIR spectrum of UA-Ch-NPs suggest the functional interaction between UA and Ch. Two small peaks at 1600 and 1700 cm^–1^ in the spectrum of UA-Ch-NPs are related to the stretching vibrations of C=O and C-N carbonyl groups associated with the formation of an amide bond between carboxylic acid, ursolic acid, and chitosan amine [22]. The peak at 1550 cm^–1^ is attributed to the N-H bending vibrations and the wide peak around 3400 cm^–1^ is related to the O-H stretching vibrations at the Ch surface. The emergence of these peaks in the spectrum of UA-Ch-NPs and the absence of the characteristic peaks of UA confirm the binding interaction between Ch and UA through the formation of amide bonds.

The results of DLS and zeta potential analysis of UA-Ch-NPs are presented in Figure 1c. Accordingly, the average particle size of UA-Ch-NPs is 258 nm, and its polydispersity index (PDI) is 0.409. Based on the results, the zeta potential of UA-Ch-NPs is +40.1 mV, which is associated with positively charged amine groups on the Ch surface. The XRD patterns of UA and UA-Ch-NPs are shown in Figure 1d. The characteristics peaks associated with UA were observed at 2θ values of 5.15, 8.81, 10.30, 14.50, 16.43, 21.40, and 27.22. These characteristic peaks were also observed in the diffraction pattern of the UA-Ch-NPs indicating the loading of UA on Ch nanoparticles. Ch usually shows a broad diffraction peak at 2θ = 22.6 ° which is also seen in the UA-Ch-NPs diffraction along with some peaks related to UA. The presence of similar characteristic peaks related to UA and UA-Ch-NPs was reported in a previous research [24].

### 3.2. Antibacterial potency of UA-Ch-NPs

**Figure 2:**
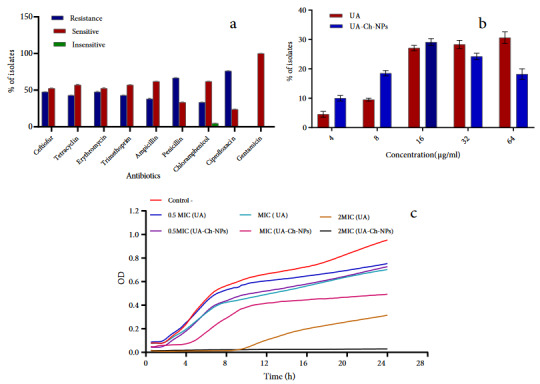


Antibiotic sensitivity of 21 *S. aureus* strains, isolated from bovine mastitis, showed different levels of antimicrobial susceptibility patterns. The antimicrobial resistance (AMR) of *S. aureus* isolates is shown in Figure 2a. Generally, the high resistance or low susceptibility to antibiotics was observed with penicillin and ciprofloxacin where 66.2% and 76.2% of the isolates were resistant, respectively. Gentamicin revealed the most effective antibiotic effect, and all the *S. aureus* strains were sensitive to this antibiotic (100% sensitive). The MIC value for UA and UA-Ch-NPs were 64 and 32 µg/mL, respectively (Figure 2b), revealing that the growth-inhibitory effects of UA-Ch-NPs were more than those of UA. Results also showed that *S. aureus* was sensitive to UA and UA-Ch-NPs at MIC, 0.5 MIC, and 2 MIC concentrations (Figure 2c). 

### 3.3. Antibiofilm effects of UA-Ch-NPs

**Figure 3:**
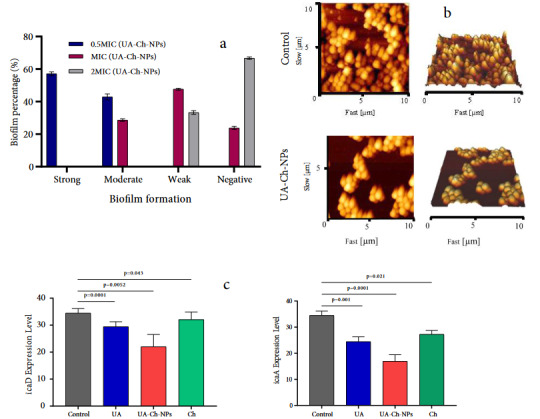


To determine the effects of UA and UA-Ch-NPs on biofilm formation by* S. aureus*, 21 strains isolated from bovine mastitis were cultured. As determined by microtiter plate assay (OD_640_), among the 21 isolates, biofilm-producing bacteria were classified as moderate (5 isolates) and strong (16 isolates). After 24 h of incubation of the bacteria with UA and UA-Ch-NPs, biofilm formation reduced drastically (Figure 3a). A number of studies have indicated a potential effect of bacterial hydrophobicity on bacterial adhesion to host tissues and biofilm formation. Therefore, cell surface hydrophobicity (CSH) and biofilm formation are significantly correlated [24,25]. Three types of hydrophobicity were identified: weak (< 30%, hydrophilic), moderate (> 30%–70%), and strong (> 70%). In this study, among the 21 isolates of bacterial strains, 62% had strong CSH, and the rest exhibited moderate hydrophobicity. As summarized in Table, among the *S. aureus* isolates, most strains were strongly hydrophobic. To further study the antibiofilm characteristics of UA-Ch-NPs, AFM analysis was used for the investigation of the interaction between the bacterial cells and the nanoparticles. AFM images of *S. aureus* showed a significant difference between biofilm formation of the control sample (*S. aureus* without treatment) and UA-Ch-NPs treatment (Figure 3b). Several research studies showed that the icaD and icaA genes of *S. aureus* have a key role in the synthesis of polysaccharide intercellular adhesion from poly-N-acetylglucosamine contributing to biofilm formation [26]. Our data also showed that UA-Ch-NPs led to a dramatic reduction in both icaA and icaD genes (Figure 3c). The statistical analysis showed that the results were statistically significant (p < 0.05).

**Table:** Interpretation of biofilm formation by S. aureus in response to UA and UA-Ch-NPs.

SD	Mean	ODC
0.1410	0.1046	0.0121
Average OD value	OD ≤ 0.1410.141 < OD ≤ 0.2820.282 < OD ≤ 0.5620.562 < OD	

## 4. Discussion

In the present study, UA-Ch-NPs were synthesized, and their antiinfective properties were investigated. Several analytical techniques were used to characterize the developed UA-containing nanoparticles to evaluate their suitability for the intended purpose. The TEM analysis showed that the synthesized UA-Ch-NPs had a good monodispersity with spherical morphology. The XRD analysis results confirmed the presence of UA on the Ch nanoparticles and from the FTIR results, the successful interaction of UA with the Ch nanoparticles surface was deduced. The DLS analysis of UA-Ch-NPs showed an average particle size of around 258 nm with a PDI of 0.409. The PDI is a value between 0 and 1. The closer this value is to zero, the greater is the uniformity of the particle size. The best value is around 0 to 0.2 for polymeric nanoparticles; for drug release purposes, this index should be below 0.4, and for other purposes, up to 0.7 is acceptable and shows the uniformity of the particle size [27]. Accordingly, the developed nanoparticles with a PDI of around 0.41 were suitable for the drug delivery purpose. The hydrodynamic diameter obtained from the DLS analysis was 258 nm, which was different from that obtained from the TEM analysis in a dry state (vacuum). This difference in particle size between DLS (258 nm) and TEM (75 nm) is attributed to the positive zeta potential of these nanoparticles that are strongly covered by the water molecules while being dispersed in aqueous solution for DLS analysis and show a large hydrodynamic diameter compared to that obtained from TEM analysis in dry state. The obtained diameter for the synthesized UA-Ch-NPs in this study is in the middle range compared to the other studies on UA-loaded nanocarriers. For instance, an average particle size of 330 nm was reported for UA-loaded Ch/poly (lactic acid) (PLA) nanoparticles [28], while another study reported a particle size of around 100–200 nm (an average of 120 nm) for UA-loaded Ch nanoparticles [10]. There is not a consensus among researchers about the suitable particle size to be absorbed by the intestinal mucosa. Some investigations have reported that particles with the size 130–950 nm are suitable to be absorbed by intestinal M cells [29]. The oversized pores of tumor microvessels varies from 100–1200 nm [30], hence, nanoparticles with a size of about 250 nm size could enter the broad fenestrations of the tumor tissues, while they are not able to enter the vessels of the normal endothelium which are narrow, approximately 5–10 nm [23]. Hence, the particle size obtained in this study has a relatively suitable size for drug delivery applications.

The zeta potential of the prepared nanoparticles was +40.1 mV demonstrating their high stability and the positive charge of the particles, which provide an effective system for drug delivery due to being taken up by proliferating cells. This effect was confirmed by previous studies [31]. The binding of nanoparticles to the cell membrane is affected by the charge of the NPs. Since cellular surfaces are dominated by negatively charged sulphated proteoglycans molecules that are highly anionic, the interaction between the proteoglycan and the nanoparticle shell (positively charged) is ionic, so the higher surface charge of the nanoparticles results in a stronger attachment of the nanoparticles to the cell membrane and higher adsorption of the nanoparticles [32]. Antonio et al. [28] also reported that by the incorporation of Ch to the surface of UA-loaded PLA nanoparticles, the zeta potential increased from –25 to +28 mV because of the amino groups of the Ch molecules, which facilitated the interaction of the nanoparticles with biological membranes.

In vitro studies demonstrated the antiinfection activity induced by UA-Ch-NPs. The antimicrobial effects of UA-Ch-NPs on the growth of *S. aureus* and its biofilm formation ability were observed. While *S. aureus* was resistant to antibiotics, it showed high susceptibility to UA and UA-Ch-NPs. In a study by Wu et al. [33], the stable antibacterial effect of triterpene derivatives such as UA was also demonstrated. In addition, the reduction of the MIC value for UA-Ch-NPs compared to UA is an indication of the higher growth-inhibitory effects of UA-Ch-NPs, which is confidently associated with the presence of Ch. It has been reported that Ch derivatives can interact with microbial cell membranes and ultimately decrease membrane integrity [34]. Qi et al. [35] also reported that Ch-NPs act against *S. aureus* by inhibiting their growth through membrane disruption and leakage of metabolites.

This study mainly aimed to produce a nanoformulation to combat *S. aureus* infection. Our findings also indicated that UA-Ch-NPs were able to inhibit bacterial growth in a concentration-dependent manner. According to Figure 2c, the developed UA-Ch-NPs prolonged the lag phase of *S. aureus* and also reduced the OD. Generally, during the lag phase, cells are depleted of metabolites and enzymes because they are adapting to their new growth conditions. In this phase, intermediates, enzymes, RNA, and other molecules are made to resume the growth. In this situation, the occurrence of a long lag phase is probable, which represents the period required for sufficient multiplication of a few mutants in the inoculum for an apparent rise in cell number [36]. 

In addition, our findings also showed that UA-Ch-NPs reduced the biofilm formation by the *S. aureus* isolates. Emerging data is trying to characterize genes engaged in biofilm formation. For example, the icaA and icaD genes have been extremely characterized before being engaged in *S. aureus* biofilm formation [36]. These genes contribute to the intercellular attachment of bacteria and biofilm formation. Our data also clearly indicated that the treatment of bacterial cells with UA-Ch-NPs significantly reduced icaA and icaD, and consequently, biofilm formation. Qian et al. [37] also reported that UA disrupted the cell membrane integrity of carbapenem-resistant *Klebsiella pneumoniae*, and strong inhibitory influences against the expression of biofilm-related genes and biofilm formation were reported.

## 5. Conclusions 

The current study managed to produce UA-Ch-NPs successfully. According to the TEM analysis, the synthesized nanoparticles were uniform in size with a good monodispersity. Moreover, the FTIR analysis results showed the interaction between functional groups of UA and Ch and thereby the successful loading of UA on the nanoparticles. The XRD results also showed the characteristic peaks associated with UA in UA-Ch-NPs. According to the results, a decrease in the MIC value of UA-Ch-NPs in comparison with UA against *S. aureus *revealed the enhanced inhibitory effect of the UA-loaded nanoparticles due to the presence of Ch nanoparticles. This pharmaceutical agent reduced the growth of *S. aureus* and its biofilm formation, which was also confirmed by the AFM analysis. The treatment of bacterial cells with UA-Ch-NPs significantly decreased the expression of icaA and icaD genes which are engaged in biofilm formation. The results of this study indicated that UA-Ch-NPs could serve as an antibiotic agent, however, this needs to be confirmed by further research in the future. 
